# Global analysis of DNA methylation in hepatocellular carcinoma by a liquid hybridization capture-based bisulfite sequencing approach

**DOI:** 10.1186/s13148-015-0121-1

**Published:** 2015-08-21

**Authors:** Fei Gao, Huifang Liang, Hanlin Lu, Junwen Wang, Meng Xia, Zhimei Yuan, Yu Yao, Tong Wang, Xiaolong Tan, Arian Laurence, Hua Xu, Jingjing Yu, Wei Xiao, Wei Chen, Ming Zhou, Xiuqing Zhang, Qian Chen, Xiaoping Chen

**Affiliations:** Hepatic Surgery Centre, Tongji Hospital, Tongji Medical College, Huazhong University of Science and Technology, Wuhan, 430030 Hubei China; Science & Technology Department, BGI-Shenzhen, Shenzhen, 518083 Guangdong China; Division of Gastroenterology, Department of Internal Medicine, Tongji Hospital, Tongji Medical College, Huazhong University of Science and Technology, Wuhan, 430030 Hubei China; The Newcastle upon Tyne Hospitals NHS Foundation Trust, Freeman Hospital, Freeman Road, High Heaton, Newcastle upon Tyne, NE7 7DN UK; Department of Urology, Tongji Hospital, Tongji Medical College, Huazhong University of Science and Technology, Wuhan, 430030 Hubei China; Translational Medicine Center, Tongji Hospital, Tongji Medical College, Huazhong University of Science and Technology, Wuhan, 430030 Hubei China

**Keywords:** DNA methylation, Liquid hybridization capture-based bisulfite sequencing, Hepatocellular carcinoma

## Abstract

**Background:**

Epigenetic alterations, such as aberrant DNA methylation of promoter and enhancer regions, which lead to atypical gene expression, have been associated with carcinogenesis. In hepatocellular carcinoma (HCC), genome-wide analysis of methylation has only recently been used. For a better understanding of hepatocarcinogenesis, we applied an even higher resolution analysis of the promoter methylome to identify previously unknown regions and genes differentially methylated in HCC.

**Results:**

Optimized liquid hybridization capture-based bisulfite sequencing (LHC-BS) was developed to quantitatively analyze 1.86 million CpG sites in individual samples from eight pairs of HCC and adjacent tissues. By linking the differentially methylated regions (DMRs) in promoters to the differentially expressed genes (DEGs), we identified 12 DMR-associated genes. We further utilized Illumina MiSeq combining the bisulfite sequencing PCR approach to validate the 12 candidate genes. Analysis of an additional 78 HCC pairs on the Illumina MiSeq platform confirmed that 7 genes showed either promoter hyper-methylation (*SMAD6*, *IFITM1*, *LRRC4*, *CHST4*, and *TBX15*) or hypo-methylation (*CCL20* and *NQO1*) in HCC.

**Conclusions:**

Novel methylome profiling provides a cost-efficient approach to identifying candidate genes in human HCC that may contribute to hepatocarcinogenesis. Our work provides further information critical for understanding the epigenetic processes underlying tumorigenesis and development of HCC.

**Electronic supplementary material:**

The online version of this article (doi:10.1186/s13148-015-0121-1) contains supplementary material, which is available to authorized users.

## Background

Hepatocellular carcinoma (HCC) represents an endemic burden worldwide. Well-known risk factors associated with HCC include chronic hepatitis B virus (HBV) and hepatitis C virus (HCV) infections and toxic, metabolic, and immune-related conditions [1].

The development of HCC is a multistep process characterized by the accumulation of genetic mutations and epigenetic aberrations. Epigenetic alterations such as aberrant methylation and histone modification occur far more frequently than genetic mutations in cancers and can significantly affect the efficacy of messenger RNA (mRNA) synthesis without changing the primary DNA sequence [2]. Identification of specific DNA methylation signatures thus has great potential to generate diagnostic markers for early disease detection and further the development of therapeutic regimens.

In mammalian cells, DNA methylation occurs at the 5′ position of the cytosine ring within CpG dinucleotides, via the addition of a methyl group, to create a 5-methylcytosine (m5C). DNA methylation sites tend to cluster in regions of large repetitive sequences, called CpG Islands (CGIs) [3]. The two most common forms of aberrant CpG methylation in cancer have been widely studied, namely global hypo-methylation that causes chromosomal instability and promoter hypo- or hyper-methylation that leads to inappropriate activation of oncogenes or silencing of tumor suppressor genes (TSGs), respectively [3, 4].

A number of powerful technologies have emerged in recent years that allow high-throughput detection of genome-wide epigenetic changes in HCC, furthering our understanding of the impact of altered DNA methylation on hepatocarcinogenesis [5–11]. For instance, promoter microarray-based approaches include the methylated CpG island amplification microarray chip (MCAM-chip) that utilizes enzymatic digestion [5, 6] and the methylated DNA immunoprecipitation microarray chip (MeDIP-chip) that employs antibody pulldown [12] to enrich methylated DNA. This is followed by profiling on a promoter array and generally results in approximately 25,000 human promoters being analyzed per sample. The second approach relies on bead arrays, which are characterized by bisulfite conversion of DNA followed by the use of a microbead-based microarray. The highest throughput achieved by this technique to date was the mapping of more than 485,000 CpG sites in HCC through an Infinium 450K array [10, 11]. Studies that have utilized currently available genome-wide profiling techniques have reported numerous differentially methylated genes in HCC, including tumor suppressor genes. Although many of the genes identified in these investigations have differed, some consistencies have been reported. For example, two studies identified *KLHL35*, *PAX5*, *PENK*, and *SPDYA* to be hyper-methylated in HCC of viral etiology [6, 9], while independent studies have also found *IGFALS* [8, 13] and *MT1G* [8, 13] to be repressed by hyper-methylation in HCC. However, there still remains no general consensus as to which genes consistently show differential methylation in HCC. In part, this may be due to intra- or inter-tumor heterogeneity, differences between studies in the etiology underlying the HCC, or differences in the technique and detection sites used, highlighting the necessity for additional investigations to identify those genes that most consistently show aberrant methylation. An important limitation of previous studies is that none have been able to detect all CpG sites in the entire promoter regions and thereby map the promoter methylome of human HCC. In order to address this shortcoming, we have enhanced the coverage to include promoter regions genome-wide and attempted to identify promising methylation markers or characteristic driver genes that may not have been reported previously in HCC.

We previously developed a liquid hybridization capture-based bisulfite sequencing (LHC-BS) technique suitable for CpG methylation analysis using a massive parallel sequencer-based approach, which relies on specific capture of target regions by liquid hybridization. We demonstrated that this approach could be used to examine the human exome [14] as well as the promoter methylome [15]. In the present study, we initially performed promoter-targeted LHC-BS on eight paired HCC tissues to analyze 1.86 million CpG sites located at the promoter regions of 31,372 (91.8 %) genes. Next, high-depth RNA-sequencing was applied to search for candidate genes in HCCs that showed a negative correlation between gene expression and promoter methylation. Illumina MiSeq combining the bisulfite sequencing PCR approach was further carried out to validate these candidate genes in an additional 78 HCC tumor and non-tumor pairs. Using this approach, we confirmed that 7 genes showed altered promoter methylation in HCC, with *SMAD6*, *IFITM1*, *LRRC4*, *CHST4*, and *TBX15* exhibiting promoter hyper-methylation, and *CCL20* and *NQO1* exhibiting promoter hypo-methylation. Western blot and quantitative real time polymerase chain reaction (qRT-PCR) experiments confirmed that a total of 5 genes showed altered expression in HCC samples. Therefore, LHC-BS-based promoter methylome analysis in HCC represents an effective technique for assessing epigenetic changes across the human genome.

## Results

### The promoter methylome differentiates tumor tissue from adjacent non-tumor tissue in HCC

The clinicopathologic features of the 8 patients with HCC in this promoter-wide methylation study are described in Additional file 1: Table S1. The primary etiology of this group was HBV infection (7 of 8 patients). All patients had a single tumor and most of the primary tumors (5 of 8) had moderately differentiated histology; 6 of 8 had stage II tumor, classified using the American Joint Committee on Cancer (AJCC) TNM system.

A LHC-BS approach [14, 15] was subsequently applied to profile the promoter methylome of the 8 sample pairs. Promoters were denoted as regions from −2200 bp to +500 bp of the transcriptional start sites (TSS) [16]. Based on the hg19 reference human genome, a total of 150,407 capture probes from the Crick strand were customized, capturing 1.86 million CpG nucleotides in the promoters. Based on this design, the Watson strand can be captured, enabling a coverage of 31,372 (91.8 %) genes in the RefSeq database [15]. We obtained an average of 4.4 Gb clean data for each sample, reaching 23× read depth, of which 94.77 % were mapped to at least one genomic position, with 87.75 % mapped uniquely to the reference genome. Furthermore, 94.21 % of the uniquely mapped reads were located at the defined promoter regions (Additional file 2: Table S2). We then filtered out all the CpG sites with less than 4× coverage in the 8 paired samples. The median value of CpG coverage between the lowest (994,997) and highest (1,685,393) sample was 1.374799 million CpGs.

**Fig. 1:**
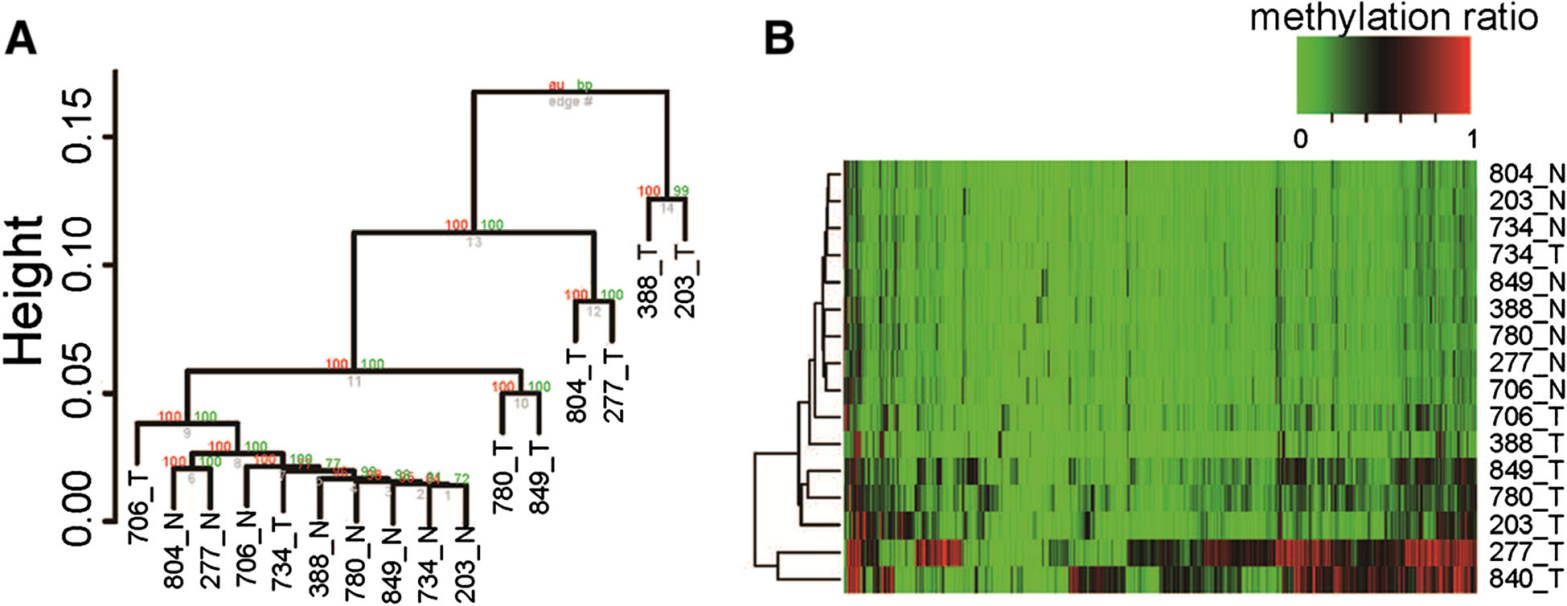
Hierarchical clustering analyses of the promoter methylomes of 8 pairs of HCC and adjacent non-tumor samples. **a** Clustering of the average DNA methylation levels of all promoters were used in the “Pvclust” algorithm. Two types of *P* values (%) on the edge of the cluster are provided: approximately unbiased (AU) *P* value and bootstrap probability (BP) *P* value, which indicate how well the cluster is supported by the data. **b** Clustering of the top 1000 CGIs containing highly variable methylations that were selected based on *P* values from a chi-square analysis. The methylation ratio was calculated as sequenced reads number of C/sequenced reads number of C + T. *Red color* indicates high methylation ratio, *black color* indicates moderate methylation ratio, and *green color* indicates low methylation ratio. *_T* denotes tumor tissue samples, and *_N* denotes non-tumor tissue samples

To identify differential methylation of CpG loci linked to HCC, we further picked 690,858 CpG sites achieving a minimum read coverage of 4× in all 16 samples and performed a hierarchical clustering analysis. Based on the average level of methylation crossing downstream 500-bp region around TSS, the tumor could be clearly separated from adjacent non-tumor by significant changes in the pattern of the promoter methylome (Fig. 1a). Principal component analysis (PCA) consistently demonstrated that HCC tissue exhibited greater variance than non-tumor tissue, and this was further confirmed by chi-square tests (Additional file 3: Figure S1A, B). These findings may suggest generalized disruption of the integrity of the methylome in HCC.

### The promoter regions of genes losing or acquiring DNA methylation show different CpG contents in HCC

To analyze the relationship between promoter DNA methylation and activity determined by the CpG content of the promoters, we applied a previously described classification of promoters as having either high-CpG content (HCP), intermediate CpG content (ICP), or low-CpG content (LCP), based on the CpG ratio, GC content, and length of the CpG-rich region [16] (Additional file 4: Figure S2A). In line with a previous report using MeDIP technology, our analysis demonstrated that genes acquiring low DNA methylation levels in tumors were mostly characterized by the presence of HCP promoters (Additional file 4: Figure S2B). We further performed hierarchical clustering analyses of CGIs, and a chi-square analysis was then applied to select the top 1000 genes containing highly variable CGI methylations based on the *P* values (Fig. 1b). In general, many of these 1000 genes had a substantially higher methylation ratio in tumor tissue than in non-tumor tissue (Fig. 1b). Interestingly, the majority of these 1000 CGIs were consistently hypo-methylated in poorly differentiated tumor (ID NO. 388) compared with moderately or well-differentiated tumors. Although one tumor (ID NO. 734) clustered closely with its adjacent tissue, we suspect that this may have been due to contamination of the tumor sample with non-tumor tissue. Overall, the data support the possibility that enriched HCPs may be responsible for inhibiting the expression of the corresponding genes.

### Comparisons of promoter CpG methylation between HCC tissue and adjacent tissue reveal differentially methylated regions and DEGs

We next applied a pair-wise comparison to reveal differentially methylated regions (DMRs). In each comparison, the sliding window strategy was used to determine if the region within the window exhibited differential methylation between tumor and non-tumor samples (Additional file 5: Material and Methods). The approach generated an average of 2972 DMRs for 16 samples, although there was variation between sample pairs, suggesting high intra-tumor heterogeneity in DNA methylation (Additional file 6: Figure S3B). However, 77 genes with one or two DMRs were found in 6 of the 8 paired samples, and 67.5 % of these DMRs were hyper-methylated in the tumor tissue (Additional file 7: Table S3).

Promoter CGI methylation has frequently been associated with silencing of gene expression. To obtain expression data from the 8 HCC pairs, we used Illumina high-throughput RNA-seq technology to assess differentially expressed genes (DEGs). After removing low quality reads, we obtained 84.55 % of reads aligned to previously annotated genes, reaching 78.13 % of mapped unique reads. Our analysis determined 18,850 genes exhibiting at least one unique read. To identify DEGs, we next performed a pair-wise comparison between tumor and non-tumor tissue using a fold change cutoff of reads per kb per million (RPKM) values larger than 2 and an FDR-adjusted *P* value less than 0.01 [17]. Using this approach, the median numbers of genes identified as DEGs for the 8 paired samples were 7019, and the majority showed down-regulated expression in HCCs (Additional file 6: Figure S3A). Only 93 DEGs were shared by 6 of the 8 paired samples (Additional file 8: Table S4).

We hypothesized that there would be a relationship between the presence of DMRs in specific promoters and the DEGs in the liver tumors. As a result, 24 genes containing DMRs in promoter regions were subsequently matched and met the selection criteria in at least 5 of the 8 sample pairs (Additional file 9: Table S5). Among these, 20 genes showed expression levels negatively associated with the DMR methylation status. These included 4 genes hypo-methylated in tumor tissue (*CLCNKA*, *BAIAP2L2*, *CCL20*, and *NQO1*) and 16 genes hyper-methylated in tumor tissue (*IFITM1*, *SMAD6*, *TBX15*, *CHST4*, *LRRC4*, *PHYHD1*, *STEAP4*, *TACSTD2*, *NPC1L1*, *THRSP*, *KCNJ10*, *PALM3*, *FAM134B*, *TMEM100*, *PM20D1*, and *GRHL2*).

### Selection of candidate genes and validation of methylation in 78 pairs of HCCs by MiSeq-BSP

We further acquired an additional 78 paired samples (of HCC and adjacent tissue) to validate the genes initially identified in the LHC-BS study. Since most of the primary tumors studied in the LHC-BS analysis had a well or moderately differentiated histology, we obtained 39 well-to-moderately and 39 moderately differentiated HCCs together with their matched adjacent tissues (Additional file 10: Table S6). The majority of the patients (96 %) in our study were male; the average age at diagnosis and treatment of HCC was 47.6 ± 10.1 years; 88 % had HBV infection. With regard to the common factors associated with HCC prognosis and recurrence, 83 % of the subjects had a single tumor, 65 % of the primary tumors were more than 5 cm in diameter, 58 % of patients had stage III tumors, and 41 % of patients had blood alpha-fetoprotein (AFP) levels greater than 4000 ng/ml. Therefore, the subjects represent a group of patients with hyper-vascular primary liver malignancy, who have a poor prognosis, associated with large tumor size as well as involvement of nearby or major vessels.

**Fig. 2:**
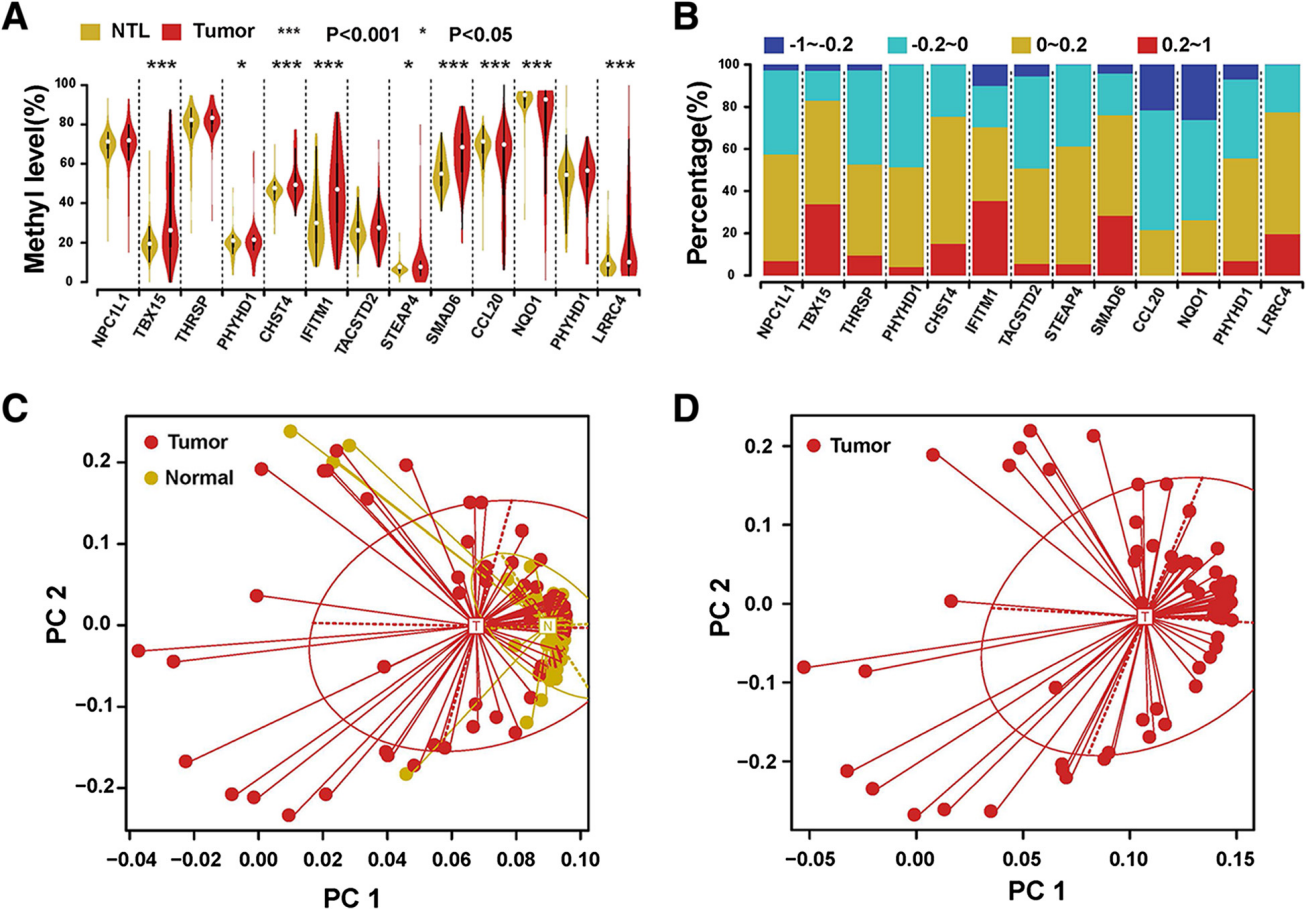
Validation of recurrent methylation changes of 12 genes. **a** Violin plots of DMR methylation levels of 12 genes in 78 pairs of HCC and non-tumor samples are shown. Differential methylation was tested using Student’s paired *t* test (***P value <0.001; *P value <0.05). **b** Distribution of altered DNA methylation of 12 genes between 78 pairs of HCC and non-tumor samples. Four categories of methylation differences between HCC and non-tumor samples are indicated. **c** Principal component analysis (PCA) of 7 validated genes in 78 pairs of HCC and non-tumor samples. **d** PCA of 7 validated genes in 78 HCC samples

By undertaking a comprehensive literature search on liver carcinogenesis, we manually selected 12 of these 20 genes showing an inverse relationship between promoter methylation and gene expression. Among these 12 genes, 10 genes up-regulated in tumors with a hyper-methylated promoter (*IFITM1*, *SMAD6*, *TBX15*, *CHST4*, *LRRC4*, *PHYHD1*, *STEAP4*, *TACSTD2*, *NPC1L1*, and *THRSP*) and 2 genes down-regulated in tumors with a hypo-methylated promoter (*CCL20* and *NQO1*) were further validated using Illumina MiSeq sequencing-based bisulfite sequencing PCR (MiSeq-BSP). Libraries for the 12 genes were prepared and individually barcoded for high-throughput pair-end sequencing using MiSeq2500 (Additional file 5: Material and Methods; Additional file 9: Table S5). Deep-sequencing of individual PCR fragments was achieved in a cost-effective way (Additional file 11: Table S7). We found that 7 genes (*IFITM1*, *SMAD6*, *TBX15*, *CHST4*, *LRRC4*, *CCL20*, and *NQO1*) showed significantly different promoter methylation levels between tumor and non-tumor tissue (*P* value < 0.001) in approximately 80 % of the 78 HCCs (Fig. 2a). In addition, 20–40 % of the examined HCCs showed a minimal difference in the mean values of 0.2 (corresponding to a 20 % difference in methylation) (Fig. 2b), indicating a highly tumor-specific promoter methylome change in these genes. We further performed supervised PCA on these 7 genes, which clearly separated tumors from non-tumors (Fig. 2c). However, the methylation status of these genes was not associated with any of the clinicopathologic findings, including histological differentiation and TNM stage (Fig. 2d).

### Validation of candidate gene expression in HCCs

**Fig. 3:**
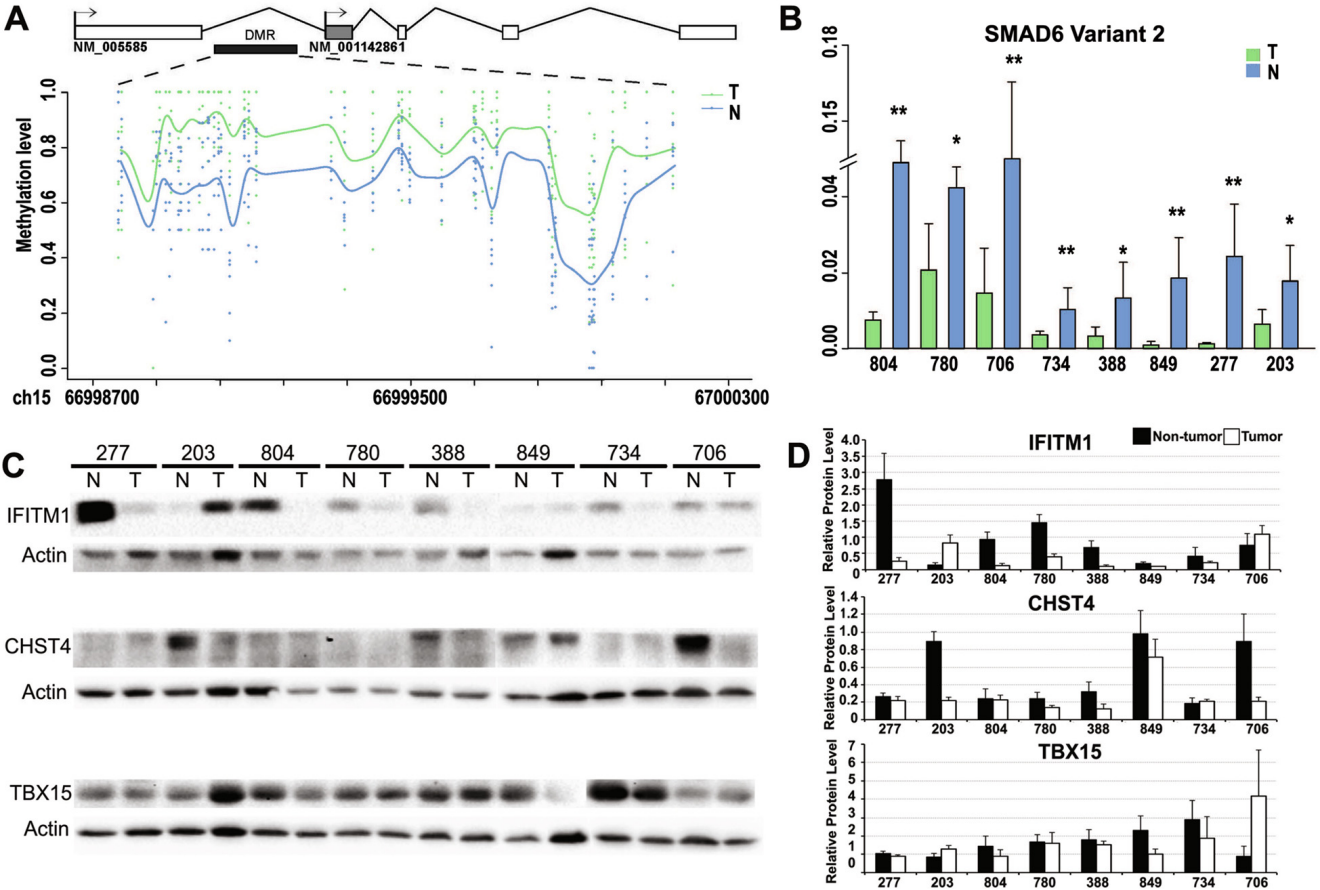
Altered expression of the candidate genes in HCC tissues. **a** Schematic of the first five exons and two TSSs of SMAD6 together with the site of the DMR. Methylation levels of CpGs within the DMR in HCC and non-tumor samples are displayed. **b** RT-PCR results of SMAD6 variant 2 in 8 pairs of HCC and non-tumor samples used in the promoter-targeted LHC-BS study. Patient IDs are shown on the *x*-axis. Data are representative of three similar experiments and displayed as mean ± SD. *P value <0.05; **P value <0.01, as evaluated using Student’s *t* test. **c** All 8 pairs of HCC samples were further validated for the protein expression of candidate genes, including *IFITM1*, *CHST4*, and *TBX15*, by Western blot analysis (*N* non-tumor tissue, *T* tumor). Patient IDs are shown above each panel. **d** Relative protein expression levels of IFITM1, TBX15, and CHST4 were normalized against actin and depicted graphically. The results are representative of three independent experiments

Among the genes that exhibited aberrant methylation in HCCs, *SMAD6* has at least two transcript variants, the full-length variant 1 (NM_005585.4) and the short variant 2 (NM_001142861.2). Genomic sequence alignment suggested that the promoter hyper-methylation observed occurred in the shorter spliced form, which lacks one in-frame exon compared with the full-length transcript, variant 1 (Fig. 3a). We further chose the primer pair specific for variant 2 and examined its expression in 8 HCC pairs assessed by the LHC-BS assay. Through qRT-PCR analysis, we confirmed reduced *SMAD6* variant 2 mRNA expressions in all examined tumors, indicating HCC-specific down-regulation of variant 2 (Fig. 3b).

Western blot analysis was further performed on the 8 HCC pairs to confirm the protein expression of the candidate genes, including *IFITM1*, *CHST4*, *TBX15*, *LRRC4*, and *NQO1*. Compared with adjacent non-tumor tissue, we observed reduced protein expression of IFITM1 in 6 of 8 tumors, lower TBX15 levels in 7 of 8 tumors, and decreased CHST4 amounts in 5 of 8 tumors (Fig. 3c, d). However, we could not detect alterations in the protein expressions of LRRC4 and NQO1 in HCC tissue (data not shown).

### Demonstration of epigenetic regulation of candidate genes transcription via demethylation assays in cell lines

**Fig. 4:**
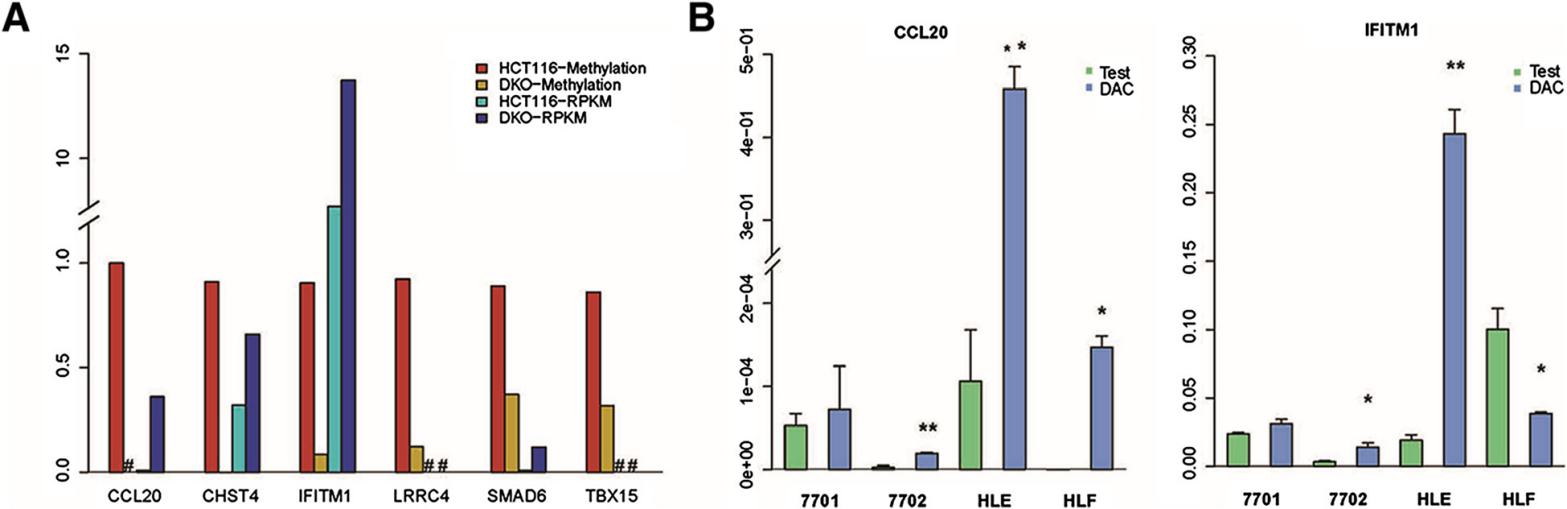
Demonstration of epigenetic regulation of candidate gene transcription. **a** Methylation and transcript expression levels of candidate genes in HCT116 and DKO cell lines are shown. **b** Demethylation assay using DAC treatment; mRNA levels of CCL20 and IFITM1 were confirmed. For RT-PCR results in **b**, the quantitative ratios were normalized to the expression of GAPDH. Data are representative of three similar experiments and displayed as mean ± SD. *P value <0.05; **P value <0.01, as evaluated by Student’s *t* test

DNMT1 and DNMT3B, which belong to the DNA methyltransferase (DNMT) family, control DNA methylation. To further evaluate the impact of promoter methylation on gene expression, we utilized two cancer cell lines, namely HCT116 wild type and HCT116^DNMT1−/− DNMT3B−/−^ double knockout (DKO) cells [18]. A total of 5 of 6 genes, including *CHST4*, *IFITM1*, *TBX15*, *LRRC4*, and *SMAD6* variant 2, showed high promoter methylation levels (>80 %) in HCT116, while more than 50 % of their methylation was lost in DKO cells as a consequence of DNMT inhibition. Correspondingly, *CCL20*, *CHST4*, *IFITM1*, and *SMAD6* variant 2 showed elevated expression in HCT116 DKO, confirmed by qRT-PCR (Fig. 4a and Additional file 12: Table S8).

For hyper-methylated or transcriptionally silenced genes, the DNA demethylating agent 5-aza-2-deoxycytidine (DAC) is known to restore gene expression [19, 20]. We further analyzed candidate gene expression upon DAC treatment in two immortalized non-tumor liver cell lines (QSG-7701 and HL-7702) and two HCC cell lines (HLE and HLF). For *CCL20*, we observed elevated expression in HCC cell lines upon treatment with DAC, but not in non-tumor cell lines, suggesting that *CCL20* methylation status may be specifically associated with HCC prognosis. In agreement with the observations made in the HCT116-DKO study, *IFITM1* expression in HCC cell lines was restored after treatment with DAC (Fig. 4b). However, *TBX15*, *LRRC4*, and *CHST4* showed no systematic difference in expression between HCC and non-tumor liver cell lines (Additional file 13: Figure S4). Nonetheless, these observations in cell lines do not exclude the possibility that the expressions of *TBX15* and *CHST4* are altered in some patients with HCC due to promoter hyper-methylation, particularly as the Western blot analysis of the 8 paired samples described above revealed that TBX15 levels were reduced in 7 of 8 tumors, and CHST4 levels were decreased in 5 of 8 tumors (Fig. 3c, d).

Taken together, our results suggest that *SMAD6* variant 2, *IFITM1*, *TBX15*, and *CHST4* may act as TSGs in HCC that are silenced by promoter hyper-methylation; meanwhile, *CCL20* may be epigenetically activated in tumor through promoter hypo-methylation, and elevated expression may be associated with a poor prognosis in HCC.

## Discussion

HCC is a genetically heterogeneous disease. The goal of genomic and epigenetic profiling efforts in studies of HCC is to identify characteristic driver genes and improve our understanding of the etiology of the disease. Promoter CpG islands with aberrant hyper-methylation are recognized as being an important mechanism for inactivation of tumor-related suppressor genes in human cancers. Although this has been extensively studied in colon cancer, with many genes identified as harboring altered methylation in their promoter CGIs [21], there is currently far less information regarding HCC. Our previous study showed that the novel LHC-BS approach is a reliable and efficient analytical platform for generating a single-base-pair resolution methylome map of promoter regions in cancer and normal cell lines [14, 15]. In the current study, we further optimized the technology to profile the promoter methylome in 8 paired HCC samples.

**Fig. 5:**
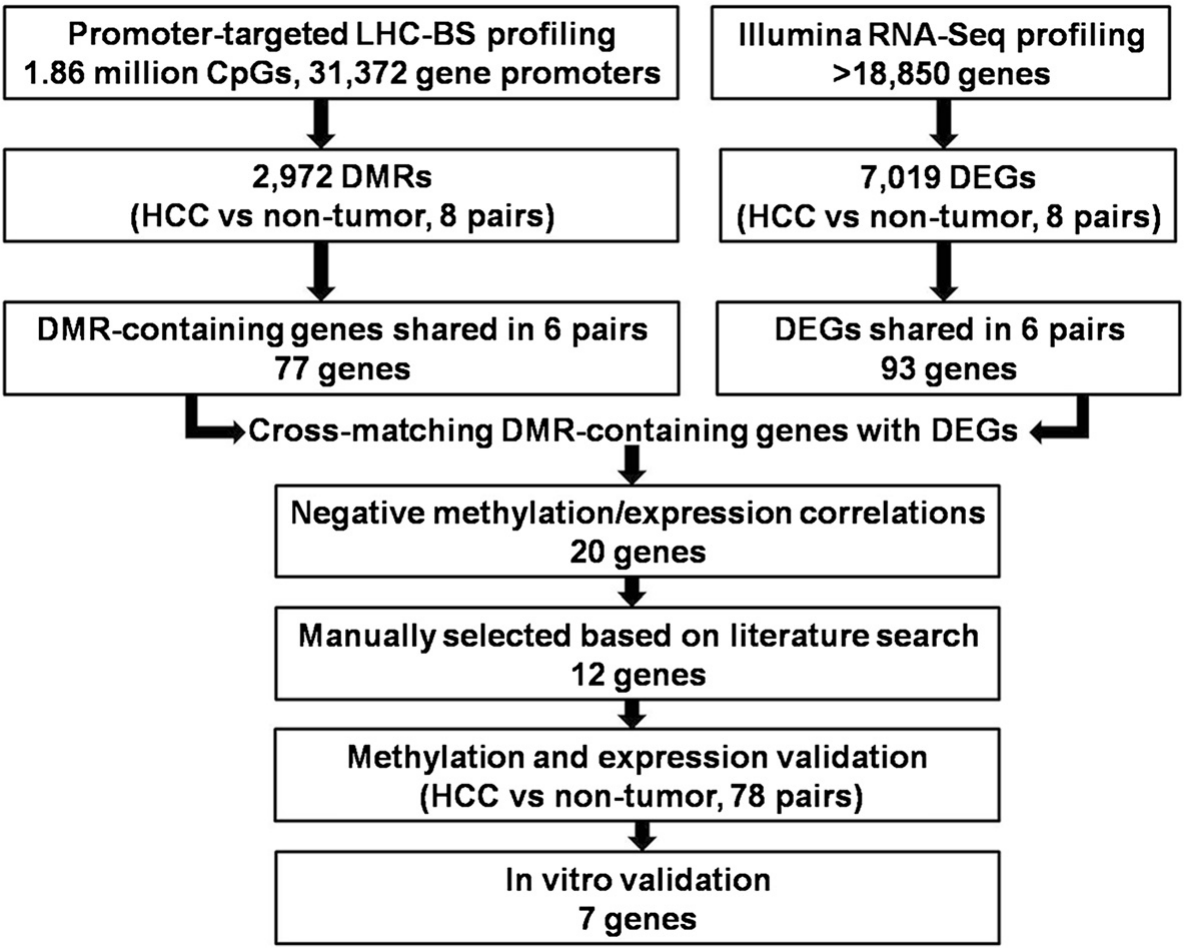
Flow chart. Eight pairs of HCC samples were used to screen for candidate methylation markers using the promoter-targeted LHC-BS approach. In HCC tissues, 2972 DMRs were determined and 77 genes with one or two DMRs were found to be common in 6 of 8 paired samples. Gene expression was analyzed using the Illumina high-throughput RNA-seq technology, and 7019 DEGs were detected. Among them, 93 DEGs were shared by 6 of the 8 paired samples. Through cross-matching DMR-containing genes with DEGs and searching the literature, 20 genes were selected for validation. Twelve candidate genes were validated for methylation and gene expression in 78 paired HCC samples. Functional validation was performed in vitro, and 7 genes were identified as candidate genes in HCC, whose altered expression may contribute to hepatocarcinogenesis

The flow chart in Fig. 5 describes the strategy we used to identify HCC-specific candidate genes. We first identified 77 genes with one or two DMRs shared by 6 of the 8 liver tumors and then detected 93 DEGs that were common to the tumors compared with non-tumor liver tissue (Additional file 7: Table S3; Additional file 8: Table S4). We further integrated these data and matched DEGs to DMRs. The approach allowed us to characterize 20 genes showing an inverse relationship between CpG methylation and transcriptional activity. Although we found greater heterogeneity in the DMR profiles among tumors as compared with non-tumors, our findings further support the idea that tumors in general have highly heterogeneous DNA methylation patterns. As a result of the limited sample size and strict criteria, we excluded low quality variants. Therefore, it should not be surprising that the current study has not yielded many overlapping candidate genes with altered promoter methylation. Indeed, to focus on genes for which differential methylation and expression could be related, we applied larger sample cohorts (78 paired HCC samples) for a further validation using Illumina MiSeq-BSP (Fig. 2). Using this approach, 7 genes were identified as undergoing tumor-specific aberrant promoter methylation in HCC, including *IFITM1*, *SMAD6*, *TBX15*, *CHST4*, and *LRRC4* with hyper-methylated promoters and *CCL20* and *NQO1* with hypo-methylated promoters.

We subsequently confirmed the correlation between promoter methylation and expression levels of the *SMAD6*, *IFITM1*, *TBX15*, *CHST4*, and *CCL20* genes (Figs. 3 and 4). In particular, we found that *IFITM1* showed significantly down-regulated expression with abnormal epigenetic regulation in HCC. *IFITM1* encodes an interferon (IFN)-induced antiviral protein that plays a key role in IFN-gamma mediated anti-proliferation either by inhibiting ERK activation or inducing p53-dependent G1 arrest [22]. The down-regulation of IFITM1 has been linked to both low-grade diffuse astrocytomas and breast cancer [23, 24]. In addition, epigenetic silencing of the IFITM1 protein has been found in other human malignancies, including gastric cancer [25]. Given that IFN-gamma plays a role during HCC immunotherapy and has a direct inhibitory effect on HCC by inducing apoptosis, future studies will be focused on understanding whether IFITM1 suppresses HCC through IFN-gamma signal transduction.

Another TSG candidate highlighted is the short transcript of *SMAD6*, variant 2, but not the full-length *SMAD6*, variant 1. *SMAD* transcription factors lie at the core of the transforming growth factor-beta (TGF-β) signaling pathway. Recent studies have suggested that differentially spliced forms of many SMAD family members are generated by genetic or epigenetic inactivation and may represent an important step in neoplastic transformation [26–28]. As noted, tumor-derived variants of SMADs often carry a mutated N- or C-domain, which inhibits the formation of homodimers or heterodimers with other SMADs, resulting in a loss of sensitivity to TGF-β cytokine family-mediated growth arrest [26, 27]. Unlike *SMAD6* variant 1, which is broadly expressed and functions as a negative regulator of bone morphogenetic protein (BMP) and TGF-β signaling [29], variant 2 has a truncated MAD homology (MH)-1 domain and is variably expressed. It has been reported that variant 2 forms non-productive heterodimers with SMAD7 in a transcriptional complex, thereby, interfering with conventional BMP/TGF-β1 signaling [30]. Additional studies are required to determine whether silencing *SMAD6* variant 2 may contribute to HCC tumorigenesis.

Treatment of HCC cell lines with DAC was associated with *CCL20* promoter hypo-methylation together with elevated expression (Fig. 4). Up-regulated *CCL20* expression is observed in many tumors including HCC [31]. The oncogenic role of *CCL20* has been characterized to be the promotion of tumor cell invasion through the up-regulation of MMP-9 in colorectal cancer cells and pancreatic adenocarcinoma [32, 33]. Our findings suggest that the presence of CCL20 in the tumor may indicate a poor prognosis of HCC. Further studies are required to completely understand the epigenetic and molecular mechanisms regulating CCL20 expression and determine whether it plays a role in metastasis in advanced HCC.

However, HBV infection is the major risk factor for HCC in China. In this study, 7 of 8 patients were infected with HBV, including tumor and adjacent liver tissues (primarily cirrhotic). The current study could not confirm an association of these genes with HBV infection and HBV-induced HCC tumorigenesis. Further studies are warranted to clarify these issues.

## Conclusions

In summary, to the best of our knowledge, this is the first report to apply promoter-targeted LHC-BS technology to assessing the promoter methylome in HCC at a single-base resolution. Our current analysis focused on differential methylation patterns in or near gene promoters. In combination with RNA-seq and gene expression data, this technique allows for the identification of promising tumor suppressor and oncogene candidates in human HCC. Our work highlights the potential of cost-efficient epigenetic approaches in the prevention and therapy of human HCC.

## Methods

### Patients and specimens

This study was approved by the Institutional Review Board of Tongji Hospital, Tongji Medical College of Huazhong University of Science and Technology (HUST) and the local ethics committee in Hubei province, China. All patients included in the study were referred for treatment at Tongji Hospital between 2008 and 2012. Written informed consent was obtained from each patient. The histological diagnosis and classification of HCC and adjacent liver tissue (primarily cirrhotic) were performed by experienced pathologists. Information about risk factors and other clinicopathologic characteristics for HCC was retrieved from medical records. Among these, HBV (hepatitis B surface antigen; HBsAg) and HCV (anti-HCV) statuses were determined by immunoassays.

### Promoter-targeted LHC-BS and RNA-seq

Promoter-targeted LHC-BS was performed as described previously [15]. Briefly, 1 μg DNA per sample was processed by fragmentation, blunt end repair, 3′adenylation, and 5′-methylcytosine index adapter ligation. Then, 250 ng DNA from each offer adapter-ligated libraries were pooled together for the liquid hybridization capture procedure. We applied capture program, bisulfite treatment, and PCR amplification based on previous protocols [15]. For RNA-seq [34], the poly(A)-containing mRNA was purified using Oligo(dT) Beads (Illumina), and this was followed by fragmentation. The converted double-stranded cDNA product was subjected to blunt-ending, dA addition to the 3′ end and adapter ligation. The adapter-ligated fragments were size selected (200 ± 20 bp) using 2 % TAE–Certified Low-Range Ultra Agarose (Bio-Rad). After purification, 15 rounds of PCR amplification were performed to enrich the adapter-ligated cDNA libraries. The LHC-BS and RNA-seq libraries were performed on an Agilent Technologies 2100 Bioanalyzer using the Agilent DNA 1000 chip kit. They were subsequently quantified on a StepOne plus qPCR, and library products were sequenced using the Illumina Hiseq2000.

### Computational processing of the next-generation sequencing data

See Additional file 5: Materials and Methods.

### 5′-aza-2′-Deoxycytidine treatment of cell lines

Two human HCC cell lines (HLE and HLF) and 2 immortalized liver cell lines (QSG-7701 and HL-7702) were cultured at 5 % CO_2_, 37 °C, and 95 % humidity in Dulbecco’s modified Eagle medium (DMEM; Gibco-Life Technologies) supplemented with 10 % fetal bovine serum (FBS; Gibco; Life Technologies), 100 units/mL penicillin, and 100 μg/mL streptomycin (Sigma-Aldrich). After growing to about 60 % confluency, cells were treated with 5 μM DAC (Sigma) for 72 h. DAC was replenished every 24 h. After 72 h, DAC-treated cells and untreated controls were harvested.

### Illumina MiSeq sequencing-based bisulfite sequencing PCR

See Additional file 5: Materials and Methods, and Additional file 11: Table S7.

### Quantitative real-time PCR

Reverse transcribed with ReverTra Ace-α-™ (Toyobo) was 1 μg of total RNA. qRT-PCR was carried out using TaqMan Universal Master Mix II with UNG on an ABI StepOne Real-Time PCR System (Applied Biosystems, USA). The relative RNA expression was calculated using the delta delta threshold cycle (ΔΔ*C*T) method and normalized to glyceraldehyde-3-phosphate dehydrogenase (GAPDH) expression. Each assay was performed in triplicate.

### Western blot

Western blot was performed with antibodies specific to IFITM1 (mouse monoclonal antibody 1:2000, Proteintech Group, USA), TBX15 (rabbit polyclonal antibody 1:1000, Aviva Systems Biology, USA), CHST4 (rabbit polyclonal antibody 1:1000, Aviva Systems Biology), LRRC4 (rabbit polyclonal antibody 1:1000, Abgent, USA), and NQO1 (mouse monoclonal antibody 1:1000, Santa Cruz Biotechnology, USA). β-actin (mouse monoclonal antibody 1:10,000, Santa Cruz Biotechnology) was used as a loading control. The expression levels of the proteins were quantified by ChemiDoc™ MP Imager Universal hood III (Bio-Rad Laboratories Inc., USA).

### Statistical analysis

All differential methylation analyses were performed using *M* values, and *β* values, ranging from 0 to 100 % methylation. Differential methylation was tested statistically using Student’s paired *t* test. CpG sites with FDR <0.05 and a within-pair methylation difference of ≥5 % were considered differentially methylated. Moderated *t* statistics with the Benjamini and Hochberg (BH) correction methods were used to compare within-pair differences of tumor and non-tumor pairs between groups, to examine whether the identified within-pair methylation discordances were group specific. Hierarchical clustering analyses were performed on the promoter methylomes of paired samples. Clustering of average DNA methylation levels of all promoters were used in the “Pvclust” algorithm. Two types of *P* values (%) on the edge of the cluster are provided: approximately unbiased (AU) and bootstrap probability (BP) *P* values. The top 1000 CGIs containing highly variable methylations were selected based on *P* values from a chi-square analysis. R and Stata statistical software (release 12.0; Stata Corporation, USA) were used for statistical analysis.

### Gene set and pathway analyses

The significance of predefined sets of CpGs, each set representing a pathway on KEGG, was analyzed by the R package GSA. GSA was applied on within-pair differences in methylation and run with 1000 permutations. An FDR cutoff of 0.1 and a *P* value cutoff of 0.05 were considered significant IPA (Ingenuity Systems, Redwood City, CA, USA), with KEGG pathways used to generate gene networks and functions in HCC tumorigenesis and development.

### Data and material availability

All raw and processed data of promoter LHC-BS and RNA-Seq have been deposited in NCBI’s Gene Expression Omnibus (GEO) with accession reference GSE55759.

## Additional files

Additional file 1: Table S1. Clinicopathology features of patients with HCC studied for genome-wide promoter methylation analysis. Additional file 2: Table S2. Data generation for LHC-BS and RNA-Seq. Additional file 3: Figure S1. Tumors are separated from adjacent non-tumors through analysis of the patterns of the promoter methylomes. A, principal component analysis (PCA) of the average methylation levels of total promoters in 8 pairs of HCCs and non-tumor samples; B, cumulative curves of –log(*P* value) from the chi-square test on intra-group variations of CpG methylation levels in HCC tumors (T) and non-tumors (N). Additional file 4: Figure S2. Promoter classification based on CpG representation. A, the histograms represent the distribution of observed versus expected CpG frequencies for all promoters, displaying the low (LCPs, red), intermediate (ICPs, green), and high (HCPs, blue) CpG content promoters; B, the proportion of the three categories of promoters. Additional file 5: Materials and Methods. Computational processing of the next-generation sequencing data; Illumina MiSeq sequencing-based bisulfite sequencing PCR (MiSeq-BSP). Additional file 6: Figure S3. Percentage of DEGs and DMR-containing genes determined in 8 paired HCC tissues. A, counts of down- and up-regulated genes in HCCs in comparison with non-tumors; B, counts of hyper- and hypo-methylated genes in HCCs in comparison with non-tumor tissue. Additional file 7: Table S3. Summary of 77 genes containing DMRs across at least 6 pairs of HCCs and non-tumors by LHC-BS. Additional file 8: Table S4. Summary of 93 DEGs shared by at least 6 paired HCC tissues. Additional file 9: Table S5. Summary of 20 genes showing negative correlation between methylation and gene expression in at least 5 out of 8 pairs of samples. Additional file 10: Table S6. Clinicopathological features of 78 Chinese HCC patients studied for validation of gene expression. Additional file 11: Table S7. Primer sequences for MiSeq-BSP and RT-PCR validation. Additional file 12: TableS8. Coverage and methylation levels of 12 genes validated by MiSeq-BSP. Additional file 13: FigureS4. RT-PCR results for TBX15, LRRC4 and CHST4 in a demethylation assay. The quantitative ratios were normalized to the expression of GAPDH. Data are representative of three similar experiments and displayed as mean ± SD. *, *P* <0.05 as evaluated by Student’s *t* test.

## Abbreviations

AFP: alpha-fetoprotein
BMP: bone morphogenetic protein
CGI: CpG Islands
DAC: 5-aza-2-deoxycytidine
DEG: differentially expressed genes
DMR: differentially methylated regions
DNMTs: DNA methyltransferases
HBV: Hepatitis B virus
HCC: hepatocellular carcinoma
HCP: high-CpG promoters
HCV: Hepatitis C virus
ICP: intermediate-CpG promoters
LCP: low-CpG promoters
LHC-BS: liquid hybridization capture-based bisulfite sequencing
MH: MAD homology
MiSeq-BSP: MiSeq sequencing-based bisulfite sequencing PCR
PCA: principal component analysis
RPKM: reads per kb per million
TSG: tumor suppressor gene
