# Supplementary figures and images for: Global analysis of DNA methylation in hepatocellular carcinoma by a liquid hybridization capture-based bisulfite sequencing approach
Source: Clin Epigenetics. 2015 Aug 21;7(1):86. doi: 10.1186/s13148-015-0121-1 (PMC4546208; doi:10.1186/s13148-015-0121-1)

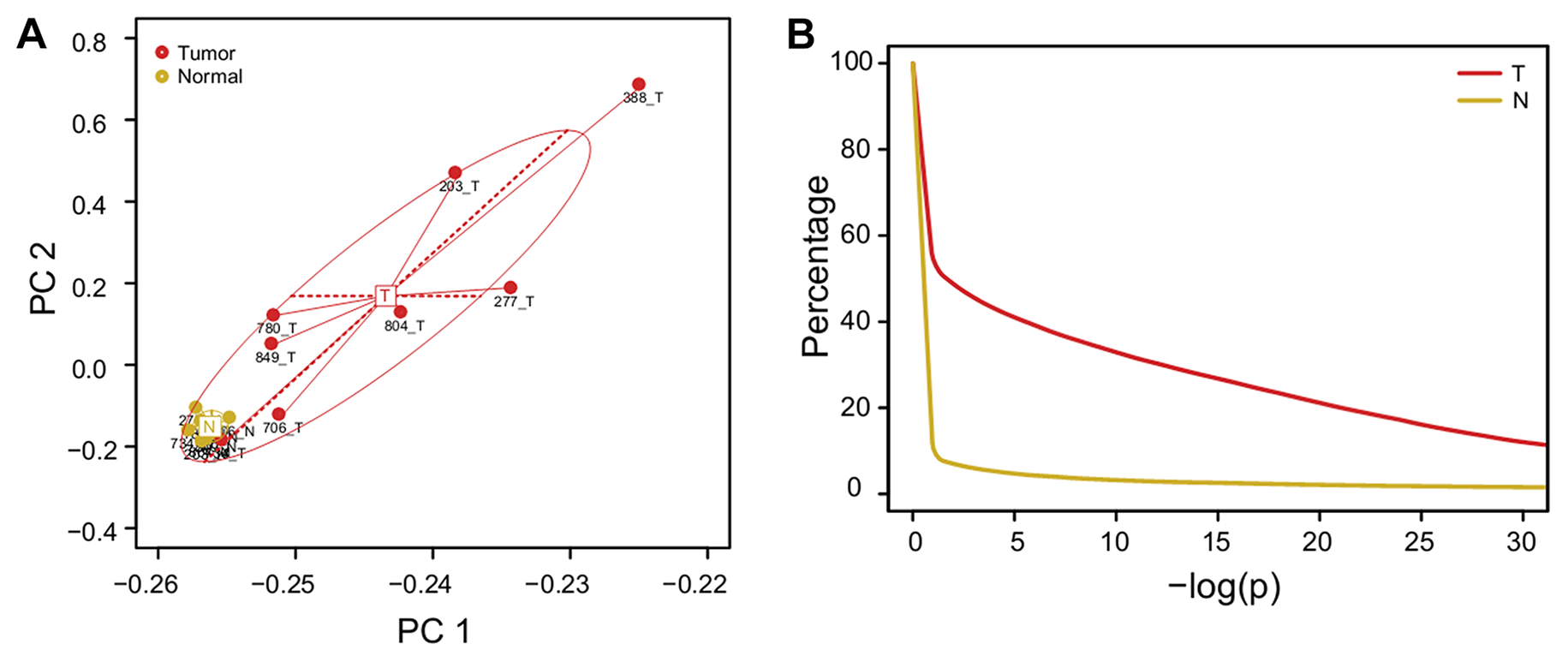

Supplement: Additional file 3: Figure S1. — Tumors are separated from adjacent non-tumors through analysis of the patterns of the promoter methylomes. A, principal component analysis (PCA) of the average methylation levels of total promoters in 8 pairs of HCCs and non-tumor samples; B, cumulative curves of –log(P value) from the chi-square test on intra-group variations of CpG methylation levels in HCC tumors (T) and non-tumors (N). [file 13148_2015_121_MOESM3_ESM.tif]

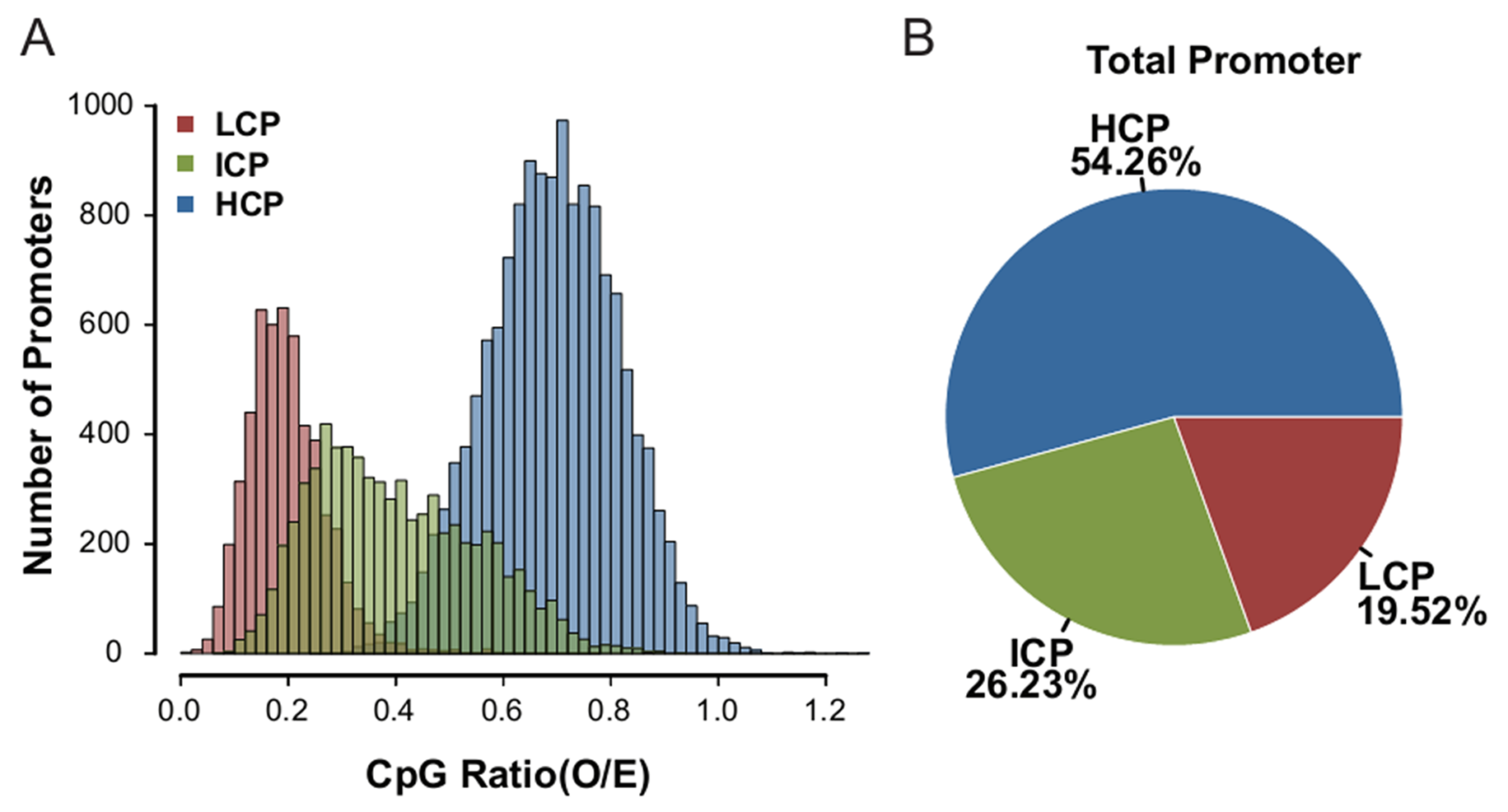

Supplement: Additional file 4: Figure S2. — Promoter classification based on CpG representation. A, the histograms represent the distribution of observed versus expected CpG frequencies for all promoters, displaying the low (LCPs, red), intermediate (ICPs, green), and high (HCPs, blue) CpG content promoters; B, the proportion of the three categories of promoters. [file 13148_2015_121_MOESM4_ESM.tif]

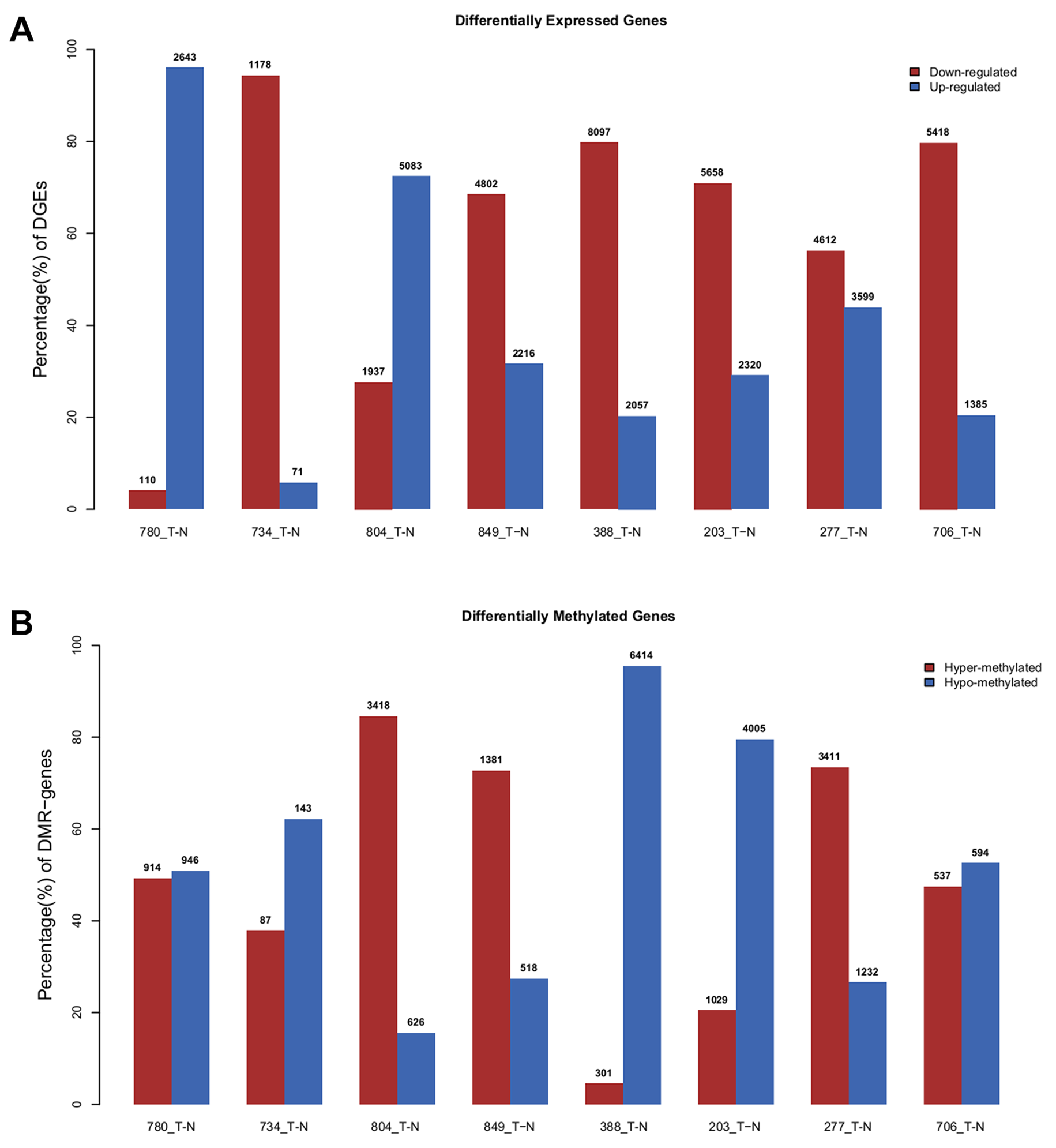

Supplement: Additional file 6: Figure S3. — Percentage of DEGs and DMR-containing genes determined in 8 paired HCC tissues. A, counts of down- and up-regulated genes in HCCs in comparison with non-tumors; B, counts of hyper- and hypo-methylated genes in HCCs in comparison with non-tumor tissue. [file 13148_2015_121_MOESM6_ESM.tif]

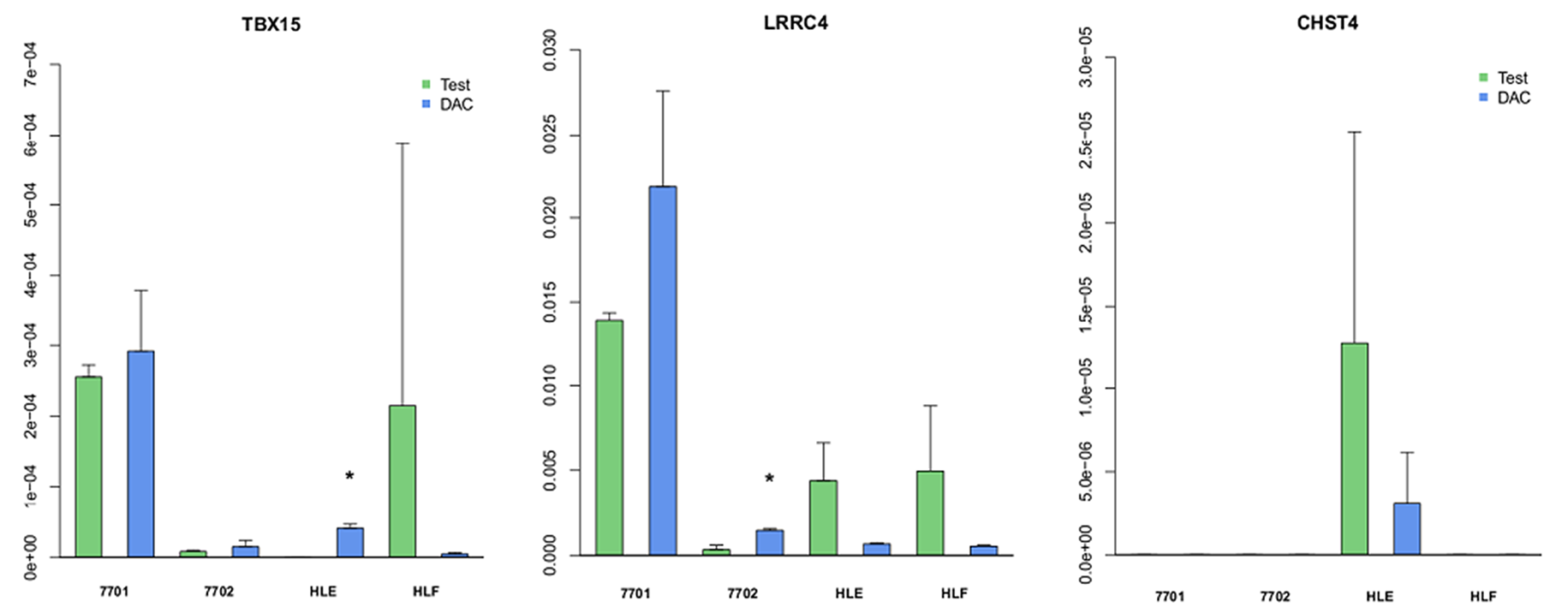

Supplement: Additional file 13: FigureS4. — RT-PCR results for TBX15, LRRC4 and CHST4 in a demethylation assay. The quantitative ratios were normalized to the expression of GAPDH. Data are representative of three similar experiments and displayed as mean ± SD. *, P <0.05 as evaluated by Student’s t test. [file 13148_2015_121_MOESM13_ESM.tif]
