# Supplementary material for: Global analysis of DNA methylation in hepatocellular carcinoma by a liquid hybridization capture-based bisulfite sequencing approach
Source: Clin Epigenetics. 2015 Aug 21;7(1):86. doi: 10.1186/s13148-015-0121-1 (PMC4546208; doi:10.1186/s13148-015-0121-1)
Supplement: Additional file 5: Materials and Methods. — Computational processing of the next-generation sequencing data; Illumina MiSeq sequencing-based bisulfite sequencing PCR (MiSeq-BSP). [file 13148_2015_121_MOESM5_ESM.docx]

**Supplementary Materials and Methods**

#### Computational processing of the next-generation sequencing data

Next-generation sequencing data were processed to remove the adapter sequences and filter out the low quality reads ([1](#_ENREF_1)). For LHC-BS data, the raw reads were aligned to the human reference genome (UCSC hg19) using BSMAP 2.73 with default parameters. The DNA methylation level of a specific cytosine was calculated as the number of reads supporting methylation divided by the total number of reads covering that cytosine. Differentially Methylated Regions (DMRs) were searched using a sliding window strategy: commonly covered CpG sites with sequencing depth ≥5X between paired samples were selected as candidate sites, and the Chi-square or Fisher test was performed to assess the significance of the methylation difference. Then, the first CpG with significantly differential methylation (P-value <0.05) was used as an initial locus of DMR, and following candidate sites were merged into a candidate DMR according to following criterion: 1) the distance between two neighboring candidate sites ≤300bp; 2) all candidate CpG sites in the candidate DMR maintain the same methylation direction (hyper- or hypo-); 3) a candidate DMR must harbor 5 or more candidate CpG sites; 4) for each of the above candidate DMRs, the Fisher test was performed to filter out the regions with a P-value >0.05 and mean methylation levels between two samples <20%.

For RNA-seq data, clean reads were mapped to a reference human gene set with no more than 2 mismatches, using SOAP aligner/SOAP2. The gene expression level was calculated by using the RPKM (Reads Per kb per Million reads) method ([2](#_ENREF_2)). Differentially expressed genes (DEGs) between two samples were identified according to a strict algorithm with FDR ≤0.001 and fold-change value ≥2; P-values were adjusted by FDR (False Discovery Rate).

#### Illumina MiSeq sequencing-based bisulfite sequencing PCR (MiSeq-BSP)

PCR primers were designed using the online MethPrimer software package (www.urogene.org/methprimer/index.html) and are listed in Additional file 10: Supplementary Table 6. Genomic DNA (500 ng) was converted using the ZYMO EZ DNA Methylation-Gold Kit™ (ZYMO) and one-tenth of the elution products were used as templates. PCR amplification was carried out with a thermal cycling program of 94°C for 1 min; 30 cycles of 94°C for 20 sec, 50~60°C for 30 sec, 72°C for 40 sec; a final 4 min incubation at 72°C. For MiSeq sequencing, PCR products (around 500bp length) of multiple genes from one sample were generated after BSP, quantified by a Qubit fluorometer with the Quant-iT dsDNA HS Assay Kit (Invitrogen, USA) and pooled together equally. 1 µg of pooled PCR products from one sample was subjected to PCR free index adapter ligation. Barcoded libraries from all samples were pooled equally and used for pair-end sequencing with 250 bp reads (PE250) using Illumina MiSeq. After sequencing, the Illumina reads were post-processed and aligned to the human reference regions (all PCR regions) using SOAP aligner (Version 2.01) with default parameters that excluded reads with more than five mismatched bases.

1. Gao F, Liu X, Wu XP, Wang XL, Gong D, Lu H, Xia Y, et al. Differential DNA methylation in discrete developmental stages of the parasitic nematode Trichinella spiralis. Genome Biol 2012;13:R100.

2. Mortazavi A, Williams BA, McCue K, Schaeffer L, Wold B. Mapping and quantifying mammalian transcriptomes by RNA-Seq. Nat Methods 2008;5:621-628.
